# Degenerative Lumbosacral Spinal Stenosis Alters Neurotrophin-3 and -4 Expression: Impact of Metabolic and Behavioral Factors

**DOI:** 10.3390/cimb47110962

**Published:** 2025-11-19

**Authors:** Małgorzata Sobańska, Dawid Sobański, Rafał Staszkiewicz, Paweł Gogol, Beniamin Oskar Grabarek

**Affiliations:** 1 Department of Neurosurgery, Szpital sw. Rafala in Cracow, 30-693 Cracow, Poland; drdsobanski@gmail.com; 2 Collegium Medicum, WSB University, 41-300 Dabrowa Gornicza, Poland; rafalstaszkiewicz830@gmail.com (R.S.); drpawelgogol@gmail.com (P.G.); bgrabarek7@gmail.com (B.O.G.); 3 Department of Neurosurgery, 5th Military Clinical Hospital with the SP ZOZ Polyclinic in Krakow, 30-901 Krakow, Poland; 4 Department of Neurosurgery, Faculty of Medicine in Zabrze, Academy of Silesia, 40-555 Katowice, Poland; 5 Department of Anesthesiology and Intensive Care, Our Lady of Perpetual Help Hospital in Wołomin, 05-200 Wołomin, Poland; 6 Department of Trauma and Orthopedic Surgery, Our Lady of Perpetual Help Hospital in Wołomin, 05-200 Wołomin, Poland; 7 Pain Treatment Clinic, Our Lady of Perpetual Help Hospital in Wołomin, 05-200 Wołomin, Poland; 8 Faculty of Medicine and Health Sciences, Andrzej Frycz Modrzewski University in Cracow, 30-705 Cracow, Poland

**Keywords:** ligamentum flavum hypertrophy, degenerative spine stenosis, neurotrophin-3 and 4, habits, lifestyle

## Abstract

Degenerative lumbosacral spinal stenosis (DLSS) is a progressive condition characterized by narrowing of the spinal canal and subsequent neural compression, often leading to chronic pain and disability. Neurotrophins, particularly neurotrophin-3 (NT-3) and neurotrophin-4 (NT-4), play essential roles in maintaining neuronal integrity and modulating nociceptive signaling; however, their involvement in DLSS and potential modulation by systemic and behavioral factors remain poorly understood. This study evaluated NT-3 and NT-4 expression in ligamentum flavum (LF) tissue from 96 patients undergoing surgical decompression for DLSS and compared the results to 85 postmortem control samples. Quantitative analyses were performed using RT-qPCR, ELISA, and immunohistochemistry. NT-3 transcript levels were markedly elevated in stenotic LF samples (fold change: 9.12 ± 0.56; *p* < 0.05), while NT-4 mRNA expression was significantly reduced (fold change: 0.33 ± 0.07; *p* < 0.05). At the protein level, both NT-3 (134 ± 5.78 pg/mL) and NT-4 (316.77 ± 8.19 pg/mL) concentrations were significantly increased compared to controls (*p* < 0.05). Although neurotrophin levels did not correlate directly with pain intensity or morphological severity, elevated NT-3 and NT-4 protein levels were significantly associated with obesity, diabetes, alcohol consumption, and tobacco use (*p* < 0.05). These findings demonstrate that NT-3 and NT-4 are differentially expressed in the ligamentum flavum of patients with DLSS and are influenced by systemic metabolic disturbances and lifestyle factors, suggesting their potential as biomarkers or therapeutic targets in degenerative spinal disease.

## 1. Introduction

Lumbosacral (L/S) spinal stenosis is a degenerative disorder resulting from progressive narrowing of the spinal canal due to hypertrophy of ligamentum flavum (LF), osteophyte formation, and intervertebral disc degeneration [1,2]. The L4/L5 level is most frequently involved, followed by L3/L4 and L2/L3. Involvement of L5/S1 and L1/L2 is less common. In advanced cases, where central or lateral recess stenosis is present, patients may experience persistent pain even at rest or during the night, and it may worsen with activities such as coughing or sneezing [3]. These structural changes lead to neural compression and ischemia, manifesting clinically as neurogenic claudication and radicular pain [4].

While the structural causes of L/S stenosis are well established, recent studies suggest that biochemical and molecular changes also play an important role in its development [5,6]. In particular, neurotrophic factors have emerged as key players in pain signaling, nerve regeneration, and inflammatory modulation [7]. These factors not only support neuronal survival but also influence local inflammatory processes and pain perception. Investigating molecular changes in affected spinal tissues, such as the LF, could deepen our understanding of disease mechanisms and help identify potential molecular targets for treatment focused on both symptom relief and neural protection [8].

Neurotrophic factors are a group of signaling proteins essential for the development, maintenance, and repair of the nervous system [9]. Among them, neurotrophins play a central role in regulating neuronal survival, synaptic plasticity, and pain processing. Beyond their classic roles during neurodevelopment, these molecules are also actively involved in modulating adult neural function and responding to injury or inflammation. Changes in the expression or activity of neurotrophins have been implicated in various chronic pain conditions, including radiculopathy and neuropathic pain syndromes [10,11].

In the context of spinal stenosis, the mechanical compression of neural tissues may trigger local inflammatory responses and alter neurotrophin signaling [12,13,14]. This can contribute to heightened pain sensitivity, altered nerve function, and impaired tissue recovery [15]. Therefore, examining neurotrophin expression in affected spinal structures may provide insight into the molecular basis of pain and dysfunction in degenerative spine disease [11].

Among the neurotrophins, neurotrophin-3 (NT-3) and neurotrophin-4 (NT-4) are of particular interest [16]. NT-3 primarily signals through its high-affinity tropomyosin receptor kinase C (TrkC) and plays a crucial role in the maintenance of proprioceptive neurons and spinal cord circuitry. It supports axonal growth and regeneration and modulates sensory neuron function. In addition to TrkC-mediated signaling, NT-3 can also bind to the low-affinity p75 neurotrophin receptor (p75NTR), which acts as a co-receptor that fine-tunes neurotrophic signaling. Depending on the cellular context, p75NTR can modulate Trk activity and influence the balance between neuronal survival and apoptosis. NT-4, in turn, binds predominantly to the tropomyosin receptor kinase B (TrkB) receptor and exhibits partial functional overlap with brain-derived neurotrophic factor (BDNF), particularly in promoting neuronal survival, synaptic maintenance, and plasticity [17,18,19,20,21].

Importantly, lifestyle factors and comorbidities such as chronic alcohol consumption, smoking, and glycemic disorders (e.g., diabetes mellitus) are known to influence both neural health and inflammatory status [22,23,24,25]. These factors can alter neurotrophin levels, impair microvascular circulation, and exacerbate neural injury, potentially intensifying symptoms of spinal stenosis [12,13,14].

Chronic alcohol intake and tobacco smoking are known to influence neurotrophin synthesis and degradation through oxidative stress and cytokine-mediated pathways. Alcohol-induced elevation of reactive oxygen species (ROS) and suppression of antioxidant defenses can upregulate NT-3 as a compensatory neuroprotective response, while prolonged exposure may disrupt NT-4/TrkB signaling and neuronal repair mechanisms [22,23,24]. Similarly, nicotine and other tobacco constituents modify NT-3 and NT-4 expression via activation of NF-κB and MAPK cascades, contributing to neuronal inflammation and altered pain perception [25,26]. In diabetes and impaired glucose metabolism, chronic hyperglycemia triggers advanced glycation end-products (AGEs) and inflammatory cytokines that reduce neurotrophic support by downregulating NT-4 and TrkB expression and altering axonal regeneration [27,28,29]. These mechanisms justify investigating NT-3 and NT-4 modulation in the context of lifestyle and metabolic disturbances among patients with degenerative spinal pathology. Neurotrophins directly participate in nociceptive signaling by sensitizing peripheral neurons and modulating central pain pathways via TrkB/TrkC receptor activation and Mitogen-Activated Protein (MAPK) Kinase–Nuclear Factor kappa-light-chain-enhancer of activated B cells (NF-κB) crosstalk [30,31]. Hence, assessing their relationship with perceived pain intensity may reflect functional consequences of altered neurotrophic signaling in degenerative spinal tissue.

The aim of the study was to evaluate the expression levels of NT-3 and NT-4 in the LF tissue of patients with degenerative lumbosacral spinal stenosis (DLSS), and to investigate how their expression is influenced by key clinical and lifestyle-related factors, including chronic alcohol consumption, smoking, and glycemic disorders.

## 2. Materials and Methods

This research builds upon our prior investigations [12,13,14,21]. This research was designed as a cross-sectional case–control study, comparing patients undergoing surgery for DLSS with a control group of LF samples obtained postmortem from individuals without spinal pathology. The study was conducted at the Department of Neurosurgery, St. Raphael’s Hospital in Cracow (30-693 Cracow, Poland) and the Department of Neurosurgery, 5th Military Clinical Hospital with the SP ZOZ Polyclinic in Krakow (30-901 Krakow, Poland), after receiving approval from the Bioethics Committee of the Regional Medical Chamber of Kraków (approval no. 224/KBL/OIL/2022).

### 2.1. Study Cohort, Pain Assessment, and Surgical Protocol

The study included 96 patients diagnosed with DLSS who qualified for surgical decompression via extended fenestration and foraminotomy. The cohort consisted of 46 women (48%) and 50 men (52%), with a mean age of 68.3 ± 2.4 years. Diagnosis was established based on comprehensive clinical evaluation, physical examination, and magnetic resonance imaging (MRI) using 3 mm and 4 mm slice thickness across multiple planes to ensure precise anatomical assessment of spinal canal narrowing and LF hypertrophy.

Participants provided information regarding lifestyle factors, including cigarette smoking and alcohol consumption, through standardized self-report questionnaires; however, detailed quantitative data concerning frequency or amount of use were not collected. The presence of diabetes mellitus was verified on the basis of medical documentation and biochemical assessment, including glycated hemoglobin (HbA1c) and fasting blood glucose levels, obtained within six months prior to surgery.

Patients were eligible for inclusion if they presented with MRI-confirmed degenerative lumbar spinal stenosis, were between 18 and 80 years of age, exhibited no contraindications to surgical or internal medical treatment, and had failed to achieve satisfactory improvement after a minimum of six months of conservative therapy. All patients receiving anticoagulant medication were required to discontinue or temporarily suspend such treatment under physician supervision before surgery. Exclusion criteria included previous spinal surgery at the lumbosacral level, effective outcomes following conservative management, lack of radiologic evidence of stenosis, pregnancy or lactation, and the presence of significant endocrine or gastrointestinal disorders such as malabsorption syndromes. Patients who had used pharmacologically classified vitamin or mineral supplements within six months before surgery were also excluded.

Comorbidities, including diabetes mellitus, thyroid dysfunction, metabolic syndrome, and cardiovascular disease, were recorded because of their potential influence on neurotrophin expression. These systemic conditions may contribute to chronic inflammation, oxidative imbalance, and altered neuroplasticity, all of which could affect NT-3 and NT-4 signaling. Although the presence of these comorbidities was documented, they were not individually adjusted for in the final statistical models, which may have contributed to interindividual variability in neurotrophin expression profiles.

Pain severity at the time of surgical qualification was assessed using the 10-point Visual Analog Scale (VAS), where a score of 0 indicated the absence of pain and a score of 10 denoted the most intense pain imaginable. All participants reported moderate to severe pain (VAS ≥ 4). The distribution of pain intensity scores was as follows: 19 patients reported a pain level of 4, 22 reported 5, 23 reported 6, 9 reported 7, 8 reported between 8 and 9, and 7 reported the maximum score of 10.

All surgical procedures were performed under general endotracheal anesthesia. After a midline incision over the symptomatic segment, the paraspinal musculature was carefully retracted to expose the posterior spinal elements. The hypertrophic LF was excised using Kerrison rongeurs, followed by decompression of the dural sac and adjacent nerve roots through foraminotomy. The operative field was irrigated with sterile saline, and the wound was closed using standard multilayer techniques. All procedures were conducted under microscopic magnification to enhance visualization and precision. Patients without perioperative complications were typically discharged on the third postoperative day and underwent follow-up examination at the Neurosurgical Outpatient Clinic approximately four weeks after surgery.

### 2.2. Control Group Composition

The control group consisted of 85 deceased individuals, including 39 women (46%) and 46 men (54%), with a mean age of 49.17 ± 2.65 years. LF tissue samples were collected postmortem during organ procurement for transplantation or forensic autopsies, in full accordance with ethical and legal regulations governing tissue collection. Information regarding smoking habits, alcohol consumption, and the presence of diabetes mellitus was recorded when available from medical documentation or family interviews, although detailed quantitative data on consumption were not obtained. In cases where diabetes was noted, confirmation was based on medical records including recent laboratory assessments of glycated hemoglobin (HbA1c) and fasting glucose levels, ensuring accurate metabolic classification.

Histological verification of all control samples was performed using hematoxylin and eosin (H&E) staining to confirm the absence of degenerative, inflammatory, or fibrotic changes within the LF. Each specimen’s eligibility was independently evaluated and approved by two certified neurosurgeons to ensure morphological integrity and comparability with the surgical cohort.

Control individuals were required to be between 18 and 80 years of age and to have no documented history of spinal degeneration, neoplastic disease, or traumatic spinal injury. Additional inclusion criteria required the absence of endocrine or gastrointestinal disorders and no intake of pharmacologically classified vitamin or mineral supplements within six months preceding death. Exclusion criteria mirrored those applied to the patient cohort and included evidence of spinal degeneration or trauma, neoplastic conditions, pregnancy, lactation, and recent use of medications or supplements that could affect molecular or metabolic homeostasis.

Baseline clinical and sociodemographic characteristics of patients and controls are summarized in Table 1.

### 2.3. Sample Collection and Molecular Analysis

LF specimens were carefully rinsed with sterile saline and immediately preserved in sterile Eppendorf tubes containing RNAlater stabilization solution (Invitrogen Life Technologies, Carlsbad, CA, USA) to ensure RNA integrity and prevent degradation. All samples were subsequently stored at −80 °C until further molecular processing. To guarantee sample purity, the collected material was obtained exclusively from the LF during surgery, with meticulous avoidance of adjacent anatomical structures such as the annulus fibrosus or interlaminar connective tissue. Tissue harvesting was performed under direct intraoperative visualization, and the anatomical origin of each specimen was independently verified and confirmed by two certified neurosurgeons, Dawid Sobański and Rafał Staszkiewicz.

### 2.4. RNA Extraction and Quality Assessment

Total RNA was isolated using a modified Chomczyński and Sacchi method with TRIzol reagent (Invitrogen Life Technologies, Carlsbad, CA, USA). Tissue homogenization was performed using a T18 Digital Ultra-Turrax homogenizer (IKA Polska Sp. z o.o., Warsaw, Poland) to ensure complete cellular disruption and uniform sample processing. To eliminate potential genomic DNA contamination, the extracts were treated with DNase I, followed by purification using the RNeasy Mini Kit (Qiagen, Valencia, CA, USA) according to the manufacturer’s protocol. RNA concentration and purity were determined spectrophotometrically, and integrity was verified prior to downstream molecular analyses. The final RNA isolates were stored at −80 °C until use.

### 2.5. NT-3 and NT-4 mRNA Expression Analysis Using Real-Time Polymerase Chain Reaction Technique Preceded by Reverse Transcription (RTqPCR)

RT-qPCR was employed to determine NT-3 and NT-4 mRNA expression levels. Following verification of RNA concentration, purity, and integrity, reverse transcription was performed to synthesize complementary DNA (cDNA), which subsequently served as a template for quantitative amplification using target-specific primers (Genomed, Warsaw, Poland; Table 2). The GAPDH gene was used as an internal reference (housekeeping) control to normalize expression levels.

All reactions were carried out in triplicate in a final reaction volume of 50 µL. Amplification specificity was validated by melting curve analysis, confirming the absence of non-specific products or primer-dimer formation. Relative mRNA expression levels were calculated using the 2 ^−∆∆Ct^ method, with results expressed as fold change relative to the mean expression level in the control group (set to 1.00).

### 2.6. NT-3 and NT-4 Protein Concentration Analysis via Enzyme-Linked Immunosorbent Assay (ELISA)

Protein concentrations of NT-3 and NT-4 in LF extracts were determined using ELISA kits incorporating polyclonal antibodies specific for NT-3 (catalog no. BS-0160R) and NT-4 (catalog no. BS-0158R) (STI, Poznań, Poland). All assays were performed in accordance with the manufacturers’ protocols to ensure reproducibility and accuracy. GAPDH (Santa Cruz Biotechnology, Dallas, TX, USA) served as an internal control for protein normalization.

Detailed descriptions of the ELISA methodology, including validation procedures and assay performance characteristics, have been published previously [14,28].

### 2.7. Immunohistochemical (IHC) Analysis of NT-3 and NT-4 Expression

All immunohistochemical reactions were developed using identical DAB incubation times (5 min ± 15 s) to minimize background variability. Exposure conditions and microscope illumination were standardized using identical gain, contrast, and white-balance settings for every specimen. Images were captured at 200× magnification and stored in 16-bit TIFF format. Quantification of staining intensity was performed with ImageJ software (ImageJ bundled with Java 8) and the IHC Profiler plugin (National Institutes of Health (NIH), Bethesda, MD, USA) [33,34], providing an objective optical density score independent of subjective visual assessment. The mean DAB-positive area (%) was calculated from 15 randomly selected, non-overlapping fields per slide. All statistical analyses were based on raw, unedited images.

### 2.8. Statistical Analysis

All statistical analyses were conducted using standard statistical software. The Shapiro–Wilk test assessed data normality and Levene’s test verified homogeneity of variances. Continuous variables were expressed as means ± standard deviation (SD). Comparisons between two groups were performed using the independent-samples Student’s *t*-test, while differences among multiple subgroups were analyzed using one-way ANOVA followed by Scheffé’s post hoc test.

To control for Type I error due to multiple testing, *p*-values were adjusted using the Benjamini–Hochberg false discovery rate (FDR) correction. Only adjusted *p* < 0.05 were considered statistically significant. For regression analyses, potential multicollinearity was examined using variance inflation factor (VIF) diagnostics (VIF < 5 was accepted). The number of predictors included in multivariate models was limited to maintain an appropriate ratio between sample size and explanatory variables, minimizing the risk of model overfitting. Comparisons of mRNA and protein expression levels between groups were performed using the independent samples Student’s *t*-test, whereas differences among multiple subgroups—such as those defined by pain severity, BMI categories, or comorbidities—were analyzed using one-way ANOVA followed by Scheffé’s post hoc test.

For mRNA expression data, ΔCt values were first normalized to GAPDH and subsequently converted to relative expression ratios using the 2^−ΔΔCt^ method, with the control group serving as the calibrator (set to 1.00). Protein concentrations determined by ELISA were normalized to the mean control value for each neurotrophin, and intergroup comparisons were performed using *t*-tests or ANOVA, as appropriate.

Linear regression analysis was used to assess the predictive value of clinical variables—including age, BMI, diabetes status, smoking, and alcohol use—on NT-3 and NT-4 expression. The Pearson correlation coefficient (r) quantified the strength of linear associations between neurotrophin levels and pain intensity measured by the VAS. To evaluate the independent and cumulative influence of metabolic and lifestyle factors, participants were stratified by the number of risk factors (0, 1, or ≥2). Each variable (BMI, diabetes, smoking, alcohol use) was initially tested in univariate regression, followed by inclusion in multivariate linear models to control for confounding and to estimate the combined effects of multiple exposures.

Multiple regression models were constructed to identify independent predictors of NT-3 and NT-4 expression. Variables that did not achieve statistical significance in univariate analyses were excluded from the final multivariate models. Model fit and explanatory power were assessed using adjusted R^2^ values. Statistical significance was set at *p* < 0.05 for all tests. Additionally, two-way ANOVA was used to explore potential interaction effects between categorical factors such as sex, metabolic status, and lifestyle variables.

## 3. Results

### 3.1. Differential Expression of NT-3 and NT-4 in LF: mRNA and Protein Analysis

**Figure 1:**
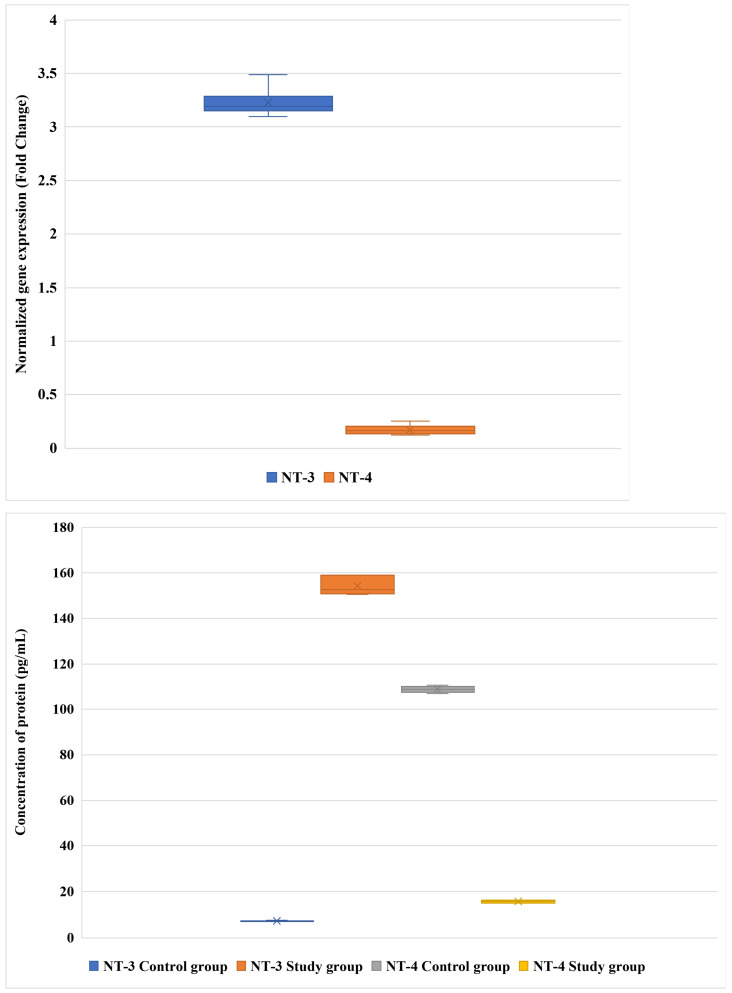
Differences in NT-3 and NT-4 expression at the mRNA and protein levels between the study and control groups.

Our results demonstrated distinct expression patterns of NT-3 and NT-4 at both the transcript and protein levels between the control and patient groups. At the mRNA level, *NT-3* expression was markedly higher than that of *NT-4*, with mean values of 3.2-fold and 0.17-fold, respectively (Figure 1; adjusted *p* < 0.05).

At the protein level, a pronounced shift in neurotrophin balance was observed. In the control group, NT-4 protein concentration dominated, reaching 109.83 pg/mL, whereas NT-3 protein levels were markedly lower (7.31 pg/mL). Conversely, in the DLSS group, this relationship was reversed: NT-3 protein expression increased sharply to 154.19 pg/mL, while NT-4 protein levels declined to 15.67 pg/mL. The differences in both mRNA and protein expression between groups remained statistically significant after FDR adjustment (adjusted *p* < 0.05 for all comparisons) (Figure 1).

Data are presented as mean ± SD. *NT-3* is neurotrophin-3; *NT-4* is neurotrophin-4.

**Figure 2:**
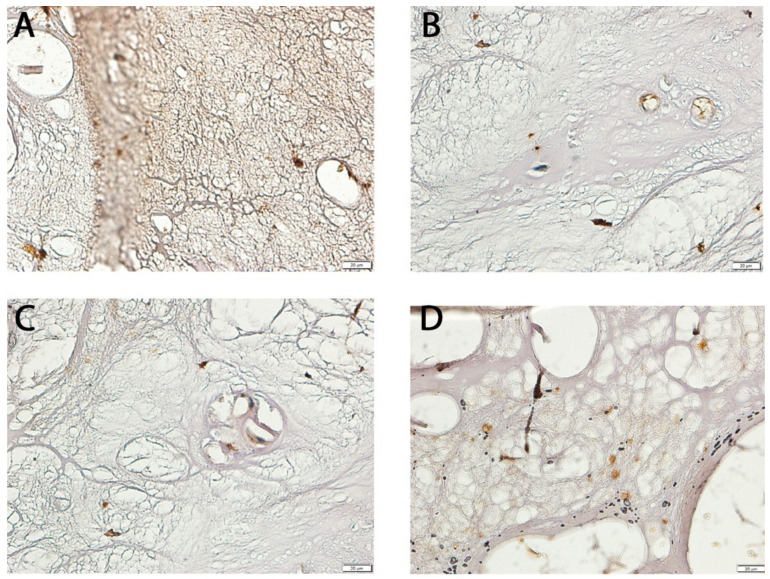
Representative immunohistochemical staining of NT-3 and NT-4 in LF tissues from study and control groups. (**A**) NT-3 expression in the test group; (**B**) NT-3 expression in the control group; (**C**) NT-4 expression in the test group; (**D**) NT-4 expression in the control group.

Visual inspection of Figure 2 is intended only as qualitative illustration. Quantitative differences confirmed by automated densitometry remained statistically significant after multiple-comparison adjustment (adjusted *p* < 0.05 for both *NT-3* and *NT-4*), ensuring that apparent contrast variations caused by DAB exposure or imaging resolution did not influence interpretation.

### 3.2. Relationship Between NT-3 and NT-4 Expression and Pain Severity

We next analyzed the relationship between NT-3 and NT-4 expression levels and the subjective intensity of pain, as evaluated by the VAS (Table 3). A clear positive correlation was observed between *NT-3* expression and increasing pain severity. Specifically, *NT-3* mRNA fold change rose progressively from 1.65 at VAS score 2 to 5.18 at VAS score 10, while corresponding protein concentrations increased from 89.03 pg/mL to 201.63 pg/mL. Statistical evaluation using one-way ANOVA confirmed the significance of these changes for both mRNA (adjusted *p* = 0.032) and protein levels (adjusted *p* = 0.041), supporting the notion that NT-3 upregulation is associated with higher pain perception in degenerative lumbosacral spinal stenosis.

In contrast, NT-4 exhibited only a modest response to pain intensity. Although protein concentrations increased from 5.13 pg/mL at lower VAS scores to 29.87 pg/mL at the highest pain levels, mRNA expression remained low and did not follow a consistent trend (adjusted *p* = 0.87). Only the differences in protein levels reached statistical significance after correction (adjusted *p* = 0.022), suggesting a limited but measurable association between NT-4 protein expression and subjective pain severity.

### 3.3. Influence of Demographic, Metabolic, and Lifestyle Factors on NT-3 and NT-4 Expression

We assessed how sex, body mass index (BMI), diabetes, tobacco use, and alcohol consumption affected neurotrophin expression in LF tissues (Table 4). NT-3 expression varied notably across subgroups. While sex had no significant impact, both BMI and diabetes status emerged as strong modulators. Obese and diabetic patients exhibited the highest NT-3 mRNA and protein levels (adjusted *p* < 0.0001). Smoking also had a marked effect, significantly elevating both transcript and protein levels of NT-3 (adjusted *p* = 0.008 and adjusted *p* = 0.042, respectively). Alcohol intake did not significantly affect NT-3 mRNA but was associated with higher protein concentrations (adjusted *p* < 0.0001).

For NT-4, expression remained consistently low, but modest differences emerged. Obesity and diabetes again correlated with higher NT-4 levels at both the mRNA and protein level (adjusted *p* = 0.002 and adjusted *p* = 0.023, respectively). Tobacco use significantly elevated NT-4 expression (adjusted *p* = 0.001 for mRNA, adjusted *p* = 0.0032 for protein), while alcohol consumption showed a minor effect only at the protein level (adjusted *p* = 0.042).

Sociodemographic analysis revealed that NT-3 and NT-4 expression patterns were not significantly influenced by sex or age but showed a clear association with higher BMI and the presence of diabetes. These findings suggest that metabolic disturbances exert a stronger modulatory effect on neurotrophin signaling than demographic variables alone.

### 3.4. Predictors of NT-3 and NT-4 Expression: Regression Modeling

#### 3.4.1. Exploratory Regression: Univariate and Multivariate Models for NT-3 and NT-4 Expression

To identify factors influencing the expression of neurotrophins, we performed univariate and multivariate regression analyses (Table 5). Initial univariate linear models using age as a predictor demonstrated no significant association with either NT-3 or NT-4 expression. However, other variables—specifically BMI, diabetes status, tobacco use, and alcohol consumption—showed strong and statistically significant correlations with both mRNA and protein levels of NT-3 and NT-4.

In the multivariable models, BMI emerged as the most consistent predictor for both neurotrophins, followed closely by diabetes. Smoking and alcohol intake also significantly influenced expression, particularly for NT-3. Gender did not exert a measurable effect in any model. All reported *p*-values were adjusted for multiple comparisons using the FDR correction, and significant predictors remained robust after adjustment. These findings suggest that metabolic and lifestyle-related variables play a pivotal role in modulating NT expression in degenerative LF tissue.

#### 3.4.2. Comparative Multivariate Regression: Control vs. Patient Cohorts

Further stratified analysis compared the control and study groups to assess whether the identified predictors operated differently across populations (Table 6). NT-3 levels were substantially elevated in the patient group and positively associated with pain intensity, BMI, diabetes, and smoking. Alcohol consumption also had a modest but significant effect. In contrast, NT-4 displayed minimal mRNA elevation but demonstrated some protein-level variation in relation to similar predictors. Gender remained non-significant across all models.

#### 3.4.3. Neurotrophin Expression and Pain: Integrative Regression with Lifestyle and Clinical Covariates

Multivariate regression analysis incorporating pain severity (VAS), BMI, smoking status, alcohol use, and diabetes revealed strong associations with NT-3 and NT-4 protein levels (Table 7). NT-3 expression was most strongly predicted by BMI (adjusted *p* < 0.0001; β = 0.41) and diabetes (adjusted *p* < 0.0001; β = 0.39), followed by smoking (adjusted *p* = 0.015; β = 0.28) and alcohol consumption (adjusted *p* = 0.028; β = 0.22). NT-4 followed a similar but slightly attenuated pattern of associations.

## 4. Discussion

Although neurotrophin expression in spinal pathologies has been examined in isolated experimental studies, most prior work has focused on NT-3 or NT-4 individually and was limited to animal models or serum-based analyses [27,29]. The present study is, to our knowledge, the first to comprehensively evaluate both neurotrophins at the transcript, protein, and histological levels directly within ligamentum flavum (LF) tissue from patients with degenerative lumbosacral spinal stenosis (DLSS). By integrating molecular findings with demographic, metabolic, and behavioral parameters—including obesity, diabetes mellitus, smoking, and alcohol consumption—this work provides novel insight into the complex interplay between systemic metabolic dysregulation and local neurotrophic signaling. We observed a striking upregulation of NT-3 expression in degenerative LF, contrasting with a significant reduction in NT-4 levels. This imbalance may reflect disrupted neurotrophic homeostasis within the local microenvironment, potentially contributing to neuropathic pain and tissue hypertrophy [35,36,37]. The consistent results obtained using RT-qPCR, ELISA, and IHC confirm the robustness of NT-3 upregulation and underscore its potential role as a molecular marker of disease severity. Notably, the strong correlations with obesity and diabetes highlight the influence of metabolic-inflammatory factors in modulating neurotrophin expression, whereas age and sex exerted minimal effects.

NT-3 and NT-4 showed divergent expression patterns: NT-3 was markedly elevated in both gene and protein analyses, while NT-4 levels were diminished. These results carry clinical implications, especially given the observed positive association between NT-3 levels and pain severity measured on the VAS [38,39].

NT-3 and NT-4 showed divergent expression patterns in degenerative LF tissue, suggesting that distinct neurotrophic mechanisms may underlie pain modulation in DLSS. The marked overexpression of NT-3 observed in our cohort may reflect a compensatory but ultimately maladaptive response contributing to aberrant neuronal remodeling and altered sensory signaling. Recent studies indicate that NT-3 can modulate nociceptive plasticity and sodium-channel expression in chronic nerve compression and inflammatory pain models, thereby influencing neuropathic pain behavior [40,41,42,43].

Elevated NT-3 levels have also been detected in patients with treatment-induced peripheral neuropathy and persistent pain after cancer therapy [41], supporting its involvement in neuroinflammatory and maladaptive sensory processes. Although direct evidence in spinal stenosis is limited, these findings collectively suggest that dysregulated NT-3 signaling may promote pathological neuroplasticity within the ligamentum flavum microenvironment in response to chronic mechanical and metabolic stress.

Increased NT-3 in degenerative LF could be a physiological response to chronic nerve compression and ischemia, activating the TrkC pathway and amplifying nociceptive transmission [44,45]. Conversely, the downregulation of NT-4 may reflect a compromised neuroprotective mechanism. NT-4 predominantly activates TrkB receptors, contributing to the maintenance of sensory neurons [46]. Reduced NT-4 levels could signal impaired regenerative capacity under chronic inflammatory and mechanical stress within the spinal canal [47]. Prior studies suggest that NT-4 activation has anti-inflammatory potential via pathways such as Tropomyosin receptor kinase B/Phosphoinositide 3-kinase/Protein kinase B/Forkhead box protein O1 (TrkB/PI3K/Akt/FoxO1), further emphasizing its protective role in neuroinflammatory conditions. The broader neurotrophin family—including NT-3 and BDNF—has been implicated in a range of neurological and neurovascular disorders, supporting their relevance as therapeutic targets [45]. Our data also reveal that NT-3 and NT-4 expression is modulated by key metabolic and behavioral variables. Patients with obesity and type 2 diabetes exhibited significantly higher NT-3 levels, which may be linked to chronic systemic inflammation and metabolic dysfunction. These findings align with earlier research indicating that cytokine imbalances and hyperglycemia can modulate neurotrophin pathways and pain thresholds [14,28,48]. Additionally, both smoking and alcohol use were associated with significant alterations in neurotrophin expression. These associations may reflect the effects of oxidative stress, altered immune signaling, and changes in neural plasticity in response to these exposures [49].

Previous experimental models support our findings. Li et al. [50] demonstrated that opioid withdrawal altered neurotrophin expression in the brain, with increased NT-4 and BDNF following naloxone treatment, while heroin suppressed their levels. Interestingly, NT-3 was upregulated in heroin-exposed animals, indicating a possible adaptive mechanism [51]. Chronic alcohol exposure has also been shown to elevate NT-3 in the hippocampus, possibly reflecting neuroadaptive responses to substance use [52]. This may explain the higher NT-3 levels observed in alcohol users in our cohort. Similarly, Requena-Ocaña et al. [53] reported that neurotrophin responses vary with educational attainment and alcohol exposure, with lower levels seen in more educated individuals, possibly due to reduced neurogenesis. Our cohort had a predominance of patients with lower education levels, which may have influenced these patterns [54].

Interestingly, although some rodent studies report reduced NT-3 in the hippocampus of smokers [55], our findings showed elevated NT-3 expression in LF samples from smoking individuals. These differences may arise from the distinct tissue-specific effects of tobacco exposure. Supporting this variability, Kimata et al. [56] noted increased NT-3 and NT-4 in tear samples from passive smokers with allergic eye disease, highlighting the role of inflammatory states in modulating neurotrophin levels. Collectively, our data suggest that lifestyle modifications, including weight control, smoking cessation, and improved glycemic regulation, could influence neurotrophic signaling in DLSS, potentially offering new avenues for symptom management and disease intervention [14,28,48].

From a surgical viewpoint, the elevated NT-3 expression in the LF of symptomatic patients may influence nerve regeneration and postoperative outcomes. Given NT-3′s role in neuronal remodeling, its preoperative levels may affect the extent of neural plasticity and repair following decompression surgery [57].

The observed correlation between NT-3 and NT-4 levels and VAS pain scores was evaluated in the context of their established roles in nociceptive transmission and central sensitization. However, pain perception is a complex, multidimensional phenomenon influenced by mechanical compression, inflammatory activity, neural remodeling, and tissue hydration. Because imaging-based stenosis grading and biochemical inflammatory parameters—such as interleukin-6 (IL-6), tumor necrosis factor-alpha (TNF-α), and C-reactive protein (CRP)—were not assessed in this study, the presented correlations should be interpreted as exploratory. Future studies integrating MRI-derived morphological indices with local cytokine profiling would enable a more precise delineation of the mechanistic link between neurotrophin expression, inflammation, and pain severity.

Several important methodological limitations should be acknowledged. The cross-sectional design precludes causal inference regarding the relationship between neurotrophin expression and disease progression. Although significant correlations were found with pain intensity, prospective longitudinal studies are required to clarify temporal dynamics and evaluate postoperative changes in NT-3 and NT-4 levels. Furthermore, lifestyle factors such as smoking, alcohol consumption, and glycemic control were based on self-reported data, which may be subject to recall bias. The absence of objective biochemical validation—for instance, HbA1c for glycemic control or cotinine levels for smoking—represents a potential source of measurement inaccuracy.

Another key methodological limitation is the exclusive use of ligamentum flavum tissue as the site of neurotrophin measurement, without inclusion of intraoperative reference tissues. During decompressive laminectomy, only the hypertrophied ligamentum flavum and, occasionally, small fragments of the facet capsule are excised; however, these adjacent structures are themselves affected by degenerative remodeling and cannot serve as healthy controls. Routine removal of paraspinal muscles is not performed; therefore, such tissues were unavailable for comparison. Nonetheless, future studies could incorporate limited samples of less-degenerated spinal soft tissues—such as the supraspinous ligament, paraspinal fascia, or facet capsule—obtained under identical surgical and handling conditions. Their inclusion would enable normalization within a shared biological microenvironment and strengthen the causal interpretation of tissue-specific neurotrophin dysregulation.

While the present study focused on gene and protein quantification, the downstream molecular pathways through which NT-3 and NT-4 contribute to ligamentum flavum hypertrophy remain incompletely defined. Both neurotrophins are known to activate TrkB/TrkC-mediated PI3K/Akt and MAPK/ERK signaling cascades that regulate fibroblast proliferation, extracellular matrix remodeling, and neuroinflammatory responses. The lack of data on signaling intermediates (e.g., phospho-Akt, ERK1/2, NF-κB) and fibrosis-related markers including transforming growth factor beta (TGF-β), and matrix metalloproteinases (MMPs) limits the mechanistic interpretation of our findings. Ongoing experiments in our laboratory aim to validate these pathways using Western blotting and immunofluorescence analyses to elucidate the molecular cascade linking neurotrophin signaling to ligamentum flavum degeneration.

Additionally, the use of postmortem control samples introduces several confounding factors. Despite stringent histological selection criteria excluding degenerative or inflammatory changes, postmortem biochemical alterations and potential RNA degradation cannot be entirely excluded [58,59,60]. The control cohort also differed substantially in age (mean 49 vs. 68 years), and neurotrophin expression is known to decline with age. Variable postmortem intervals and incomplete behavioral or metabolic data further limit the comparability of groups. Future investigations should therefore consider age-matched surgical controls or in vivo biopsy specimens, where ethically and clinically feasible, to minimize these confounding effects.

The study also lacked biochemical quantification of toxic exposure markers such as ethanol metabolites, cotinine, or carboxyhemoglobin. Consequently, associations between NT-3/NT-4 expression and lifestyle variables should be interpreted as qualitative rather than quantitative. The incomplete availability of metabolic and behavioral data for postmortem controls further limited adjustment for confounders such as undiagnosed diabetes or alcohol use. Nevertheless, the strict inclusion criteria excluding individuals with overt degenerative, metabolic, or traumatic spinal pathology helped to partially mitigate this limitation.

Finally, pain assessment relied solely on the VAS, which, although practical and widely validated, does not differentiate between neuropathic and nociceptive pain components. The use of multidimensional pain assessment tools and neurophysiological testing in future studies could refine interpretation of neurotrophin–pain relationships [61].

Despite these limitations, the present study provides a foundational molecular perspective on the role of NT-3 and NT-4 in the pathophysiology of degenerative lumbosacral spinal stenosis. Future longitudinal studies assessing postoperative neurotrophin dynamics, in conjunction with imaging, cytokine profiling, and clinical outcomes, may establish NT-3 and NT-4 as prognostic biomarkers and potential molecular targets for personalized surgical planning and perioperative management.

## 5. Conclusions

In conclusion, this study provides comprehensive evidence of differential neurotrophin expression in DLSS, demonstrating that NT-3 and NT-4 levels are closely associated with pain intensity, metabolic disturbances, and lifestyle factors. These findings deepen our understanding of the molecular mechanisms underlying DLSS-related pain and suggest that neurotrophin dysregulation may serve as a key link between systemic metabolic stress and local neuroinflammatory remodeling. From a translational perspective, NT-3 and NT-4 emerge as promising biomarkers and potential therapeutic targets. Future research should aim to elucidate the signaling pathways governing their regulation and explore targeted strategies to modulate their activity, paving the way for more individualized treatments for degenerative spinal disorders.

## Figures and Tables

**Table 1:** Clinical and sociodemographic characteristics of patients and controls.

Variable	Patients with DLSS (*n* = 96)	Controls (*n* = 85)	*p*-Value
Age (years, mean ± SD)	68.3 ± 2.4	49.2 ± 2.6	<0.001
Sex (M/F)	50/46	46/39	0.62
BMI (kg/m^2^, mean ± SD)	28.7 ± 3.9	24.5 ± 3.1	<0.01
Diabetes mellitus (%)	22.9	8.2	<0.01
Smoking (%)	37.5	28.2	0.15
Alcohol use (%)	52.1	45.9	0.38
Mean VAS pain score	6.1 ± 1.7	—	

**Table 2:** Primer Sequences for RT-qPCR Analysis of *NT-3*, *NT-4*, and *GAPDH* mRNA [32].

mRNA	Oligonucleotide Sequence
*NT-3*	Forward: 5′-CGTGGTGGCGAACAGAACAT-3′ Reverse 5′-GGCCGATGACTTGTCGGTC-3′
*NT-4*	Forward: 5′-CTGTGTGCGATGCAGTCAGT-3′ Reverse 5′-GCAGCGGGTTTCAAAGAAGT-3′
*GAPDH*	Forward: 5′-GGTGAAGGTCGGAGTCAACGGA-3′ Reverse 5′-GAGGGATCTCGCTCCTGGAAGA-3′

Forward, sensible starter; reverse, antisense primer; *GAPDH*, dehydrogenase 3-phosphoglyceraldehyde; *NT-3*, neurotrophin-3; and *NT-4*, neurotrophin-4.

**Table 3:** Expression levels of NT-3 and NT-4 in the LF of the L/S in relation to pain severity as assessed by the VAS.

Neurotrophin	Pain Intensity on the VAS	Fold Change (mRNA)	Protein Concentration (pg/mL)	ANOVA (*p*) ^b^
NT-3	2	1.65 ± 0.21	89.03 ± 2.65	0.032 ^a^ 0.041 ^b^
	3	1.87 ± 0.18	95.16 ± 2.87	
	4	2.21 ± 0.43	133.18 ± 3.91	
	5	2.31 ± 0.12	143.02 ± 5.67	
	6	2.54 ± 0.23	159.19 ± 5.91	
	7	4.01 ± 0.18	172.12 ± 5.14	
	8	4.72 ± 0.54	196.52 ± 9.82	
	9	4.51 ± 0.41	197.83 ± 9.87	
	10	5.18 ± 0.76	201.63 ± 12.34	
NT-4	2	0.19 ± 0.04	5.13 ± 0.65	0.87 ^a^ 0.022 ^b^
	3	0.31 ± 0.07	6.04 ± 0.49	
	4	0.27 ± 0.08	7.15 ± 0.91	
	5	0.13 ± 0.03	7.98 ± 0.55	
	6	0.12 ± 0.06	17.06 ± 2.36	
	7	0.11 ± 0.03	20.49 ± 2.91	
	8	0.11 ± 0.05	23.18 ± 2.45	
	9	0.14 ± 0.06	24.15 ± 3.16	
	10	0.15 ± 0.04	29.87 ± 3.81	

NT-3, neurotrophin-3; NT-4, neurotrophin-4; ^a^, *p*-value for ANOVA analysis at mRNA level; ^b^, *p*-value for ANOVA analysis at protein level. All *p*-values are FDR-adjusted (Benjamini–Hochberg method, adjusted *p* < 0.05 considered significant).

**Table 4:** mRNA and protein expression profiles of NT-3 and NT-4 in LF samples from patients with L/S stenosis, stratified by demographic, metabolic, and lifestyle factors.

Protein	Comparison		mRNA	Student’s *t*-Test ^1^ or ANOVA ^2^ (Study Group)	Protein	Student’s *t*-Test ^1^ or ANOVA ^2^ (Control Group)
NT-3	Gender	Female (*n* = 46)	3.39 ± 0.27	0.891 ^1^	145.21 ± 12.19	0.701 ^1^
		Male (*n* = 50)	3.01 ± 0.54		163.18 ± 10.12	
	BMI (kg/m ^2^)	Normal (*n* = 40)	1.00	<0.0001 ^2^	1.00	<0.0001 ^2^
		Overweight (*n* = 32)	2.02 ± 0.17		109.99 ± 8.12	
		Obesity (*n* = 24)	4.37 ± 1.01		198.39 ± 12.34	
	Diabetes	No (*n* = 46)	2.38 ± 0.52	0.001 ^1^	101.67 ± 9.12	<0.0001 ^1^
		Yes (*n* = 50)	4.02 ± 0.43		206.71 ± 18.17	
	Smoking	No (*n* = 34)	1.82 ± 0.51	0.008 ^1^	129.93 ± 11.43	0.042 ^1^
		Yes (*n* = 62)	4.57 ± 1.07		178.45 ± 8.17	
	Drinking alcohol	No (*n* = 11)	2.97 ± 0.65	0.881 ^1^	109.36 ± 9.91	<0.0001 ^1^
		Yes (*n* = 85)	3.43 ± 0.41		199.02 ± 16.91	
NT-4	Gender	Female (*n* = 46)	0.14 ± 0.03	0.812 ^1^	15.37 ± 1.09	0.753 ^1^
		Male (*n* = 50)	0.20 ± 0.06		15.97 ± 1.17	
	BMI (kg/m ^2^)	Normal (*n* = 40)	1.00	0.002 ^2^	1.00	0.023 ^2^
		Overweight (*n* = 32)	0.10 ± 0.02		12.26 ± 0.98	
		Obesity (*n* = 24)	0.23 ± 0.08		19.07 ± 1.34	
	Diabetes	No (*n* = 46)	0.12 ± 0.03	0.0122 ^1^	11.71 ± 1.65	0.0011 ^1^
		Yes (*n* = 50)	0.21 ± 0.06		19.63 ± 1.97	
	Smoking	No (*n* = 34)	0.09 ± 0.01	0.001 ^1^	12.70 ± 1.09	0.0032 ^1^
		Yes (*n* = 62)	0.25 ± 0.09		18.64 ± 2.13	
	Drinking alcohol	No (*n* = 11)	0.16 ± 0.10	0.765 ^1^	13.21 ± 2.15	0.042 ^1^
		Yes (*n* = 85)	0.18 ± 0.05		18.12 ± 2.78	

^1^, Student’s *t*-test applied for two-group comparisons; ^2^, One-way ANOVA applied for comparisons involving more than two groups; mRNA, quantitative gene expression measured by RT-qPCR. Protein: quantitative protein level measured by ELISA. LF: ligamentum flavum; L/S stenosis: lumbosacral spinal stenosis. Data are presented as means and standard deviations. All *p*-values are adjusted using the Benjamini–Hochberg false discovery rate correction (FDR-adjusted *p* < 0.05 considered significant). BMI, body mass index; NT-3, neurotrophin-3; NT-4, neurotrophin-4.

**Table 5:** Regression models identifying clinical and lifestyle variables associated with NT-3 and NT-4 expression in degenerative LF samples.

Neurotrophin	Characteristic	Expression Level	Linear Regression			Multiple Regression	
			r	R^2^	*p*-Value	Coefficient	*p*-Value
NT-3	Sex	mRNA	0.3	0.06	0.25		
		Protein	0.28	0.08	0.27		
	BMI (kg/m^2^)	mRNA	0.78	0.6	<0.0001	0.34	<0.0001
		Protein	0.79	0.64	<0.0001	0.405	<0.0001
	Diabetes	mRNA	0.7	0.75	<0.0001	0.32	0.016
		Protein	0.75	0.73	<0.0001	0.307	0.022
	Smoking	mRNA	0.86	0.44	0.002	0.47	0.023
		Protein	0.88	0.4	0.004	0.48	0.02
	Drinking alcohol	mRNA	0.55	0.38	0.025	0.23	0.027
		Protein	0.53	0.41	0.022	0.26	0.028
NT-4	Sex	mRNA	0.2	0.01	0.43		
		Protein	0.17	0.01	0.48		
	BMI (kg/m^2^)	mRNA	0.8	0.86	<0.0001	0.405	0.019
		Protein	0.77	0.8	<0.0001	0.415	0.022
	Diabetes	mRNA	0.55	0.38	<0.0001	0.316	0.018
		Protein	0.55	0.37	<0.0001	0.319	0.022
	Smoking	mRNA	0.79	0.44	0.01	0.305	0.023
		Protein	0.8	0.53	0.004	0.315	0.03
	Drinking alcohol	mRNA	0.52	0.2	0.032	0.15	0.018
		Protein	0.4	0.22	0.03	0.165	0.021

All *p*-values are adjusted using the Benjamini–Hochberg false discovery rate correction (FDR-adjusted *p* < 0.05 considered significant). NT-3, neurotrophin-3; NT-4, neurotrophin-4; BMI, body mass index; r, correlation coefficient; Variables found to be insignificant using linear regression were not included in the multiple regression model.

**Table 6:** Multivariate regression models of NT-3 and NT-4 expression in control and degenerative LF samples: effects of metabolic and behavioral factors.

Neurotrophin	Characteristic	Expression Level	Control Group		Study Group	
			Coefficient	*p*-Value	Coefficient	*p*-Value
NT-3	Gender	mRNA	-	-	-	-
		Protein	-	-	-	-
	BMI (kg/m^2^)	mRNA	0.12	0.045	0.34	<0.0001
		Protein	0.15	0.038	0.405	<0.0001
	Diabetes	mRNA	0.1	0.052	0.32	0.016
		Protein	0.14	0.048	0.307	0.022
	Smoking	mRNA	0.18	0.041	0.47	0.023
		Protein	0.2	0.039	0.48	0.02
	Drinking Alcohol	mRNA	0.09	0.060	0.23	0.027
		Protein	0.11	0.055	0.26	0.028
NT-4	Gender	mRNA	-	-	-	-
		Protein	-	-	-	-
	BMI (kg/m^2^)	mRNA	0.14	0.040	0.405	0.019
		Protein	0.16	0.035	0.415	0.022
	Diabetes	mRNA	0.11	0.050	0.316	0.018
		Protein	0.13	0.045	0.319	0.022
	Smoking	mRNA	0.17	0.039	0.305	0.023
		Protein	0.19	0.037	0.315	0.03
	Drinking Alcohol	mRNA	0.08	0.058	0.15	0.018
		Protein	0.1	0.053	0.165	0.021

All *p*-values are adjusted using the Benjamini–Hochberg false discovery rate correction (FDR-adjusted *p* < 0.05 considered significant). NT-3, neurotrophin-3; NT-4, neurotrophin-4; BMI, body mass index.

**Table 7:** Multivariate regression summary: associations between NT-3 and NT-4 protein expression and key clinical and lifestyle variables in patients.

Neurotrophin	Factor	Association with NT-3/-4 Protein (Univariate)	*p*-Value (Univariate)	Coefficient in Multivariate Model	*p*-Value (Multivariate)
NT-3	VAS Pain Score	Positive	<0.0001	0.35	<0.0001
	BMI	Positive	<0.0001	0.41	<0.0001
	Smoking	Positive	0.004	0.28	0.015
	Alcohol Consumption	Positive	0.022	0.22	0.028
	Diabetes	Positive	<0.0001	0.39	<0.0001
NT-4	VAS Pain Score	Positive	0.022	0.25	0.019
	BMI	Positive	<0.0001	0.42	<0.0001
	Smoking	Positive	0.01	0.3	0.02
	Alcohol Consumption	Positive	0.032	0.18	0.027
	Diabetes	Positive	<0.0001	0.37	<0.0001

NT-3, neurotrophin-3; NT-4, neurotrophin-4; BMI, body mass index.

## Data Availability

The raw data supporting the conclusions of this article will be made available by the authors on request.

## Abbreviations

The following abbreviations are used in this manuscript:

AGE	Advanced Glycation End-product
Akt	Protein Kinase B
ANOVA	Analysis of Variance
BDNF	Brain-Derived Neurotrophic Factor
BMI	Body Mass Index
COL1A1	Collagen Type I Alpha 1 Chain
CRP	C-Reactive Protein
DAB	3,3′-Diaminobenzidine
DLSS	Degenerative Lumbosacral Spinal Stenosis
DNA	Deoxyribonucleic Acid
DRG	Dorsal Root Ganglion
ELISA	Enzyme-Linked Immunosorbent Assay
ERK	Extracellular Signal-Regulated Kinase
FDR	False Discovery Rate
FoxO1	Forkhead Box Protein O1
GDNF	Glial Cell Line-Derived Neurotrophic Factor
GAPDH	Glyceraldehyde 3-Phosphate Dehydrogenase
H&E	Hematoxylin and Eosin
HbA1c	Glycated Hemoglobin
IHC	Immunohistochemistry
IL-6	Interleukin-6
LF	Ligamentum Flavum
L/S	Lumbosacral
MAPK	Mitogen-Activated Protein Kinase
MAPK–NF-κB	Mitogen-Activated Protein Kinase–Nuclear Factor kappa-light-chain-enhancer of activated B cells (crosstalk pathway)
MMP	Matrix Metalloproteinase
MRI	Magnetic Resonance Imaging
mRNA	Messenger Ribonucleic Acid
NF-κB	Nuclear Factor kappa-light-chain-enhancer of activated B cells
NGF	Nerve Growth Factor
NT-3	Neurotrophin-3
NT-4	Neurotrophin-4
p75NTR	p75 Neurotrophin Receptor (Tumor Necrosis Factor Receptor Superfamily Member 16)
PCR	Polymerase Chain Reaction
pg/mL	Picograms per Milliliter
PI3K	Phosphoinositide 3-Kinase
PI3K/Akt/FoxO1	Phosphoinositide 3-Kinase/Protein Kinase B/Forkhead Box Protein O1 signaling axis
RNA	Ribonucleic Acid
ROS	Reactive Oxygen Species
RT-qPCR	Reverse Transcription Quantitative Polymerase Chain Reaction
SD	Standard Deviation
TGF-β	Transforming Growth Factor Beta
TNF-α	Tumor Necrosis Factor-alpha
TrkA	Tropomyosin Receptor Kinase A
TrkB	Tropomyosin Receptor Kinase B
TrkC	Tropomyosin Receptor Kinase C
VAS	Visual Analog Scale
µL	Microliter
µm	Micrometer

## References

[1] Kahere M., Hlongwa M., Ginindza T.G. (2022). A Scoping Review on the Epidemiology of Chronic Low Back Pain among Adults in Sub-Saharan Africa. Int. J. Environ. Res. Public Health.

[2] Knezevic N.N., Candido K.D., Vlaeyen J.W., Zundert J.V., Cohen S.P. (2021). Low Back Pain: Epidemiology, Mechanisms, and Treatment. Proceedings of the Lancet-Seminar Series.

[3] Covaro A., Vilà-Canet G., De Frutos A.G., Ubierna M.T., Ciccolo F., Caceres E. (2016). Management of Degenerative Lumbar Spinal Stenosis: An Evidence-Based Review. EFORT Open Rev..

[4] Deer T., Sayed D., Michels J., Josephson Y., Li S., Calodney A.K. (2019). A Review of Lumbar Spinal Stenosis with Intermittent Neurogenic Claudication: Disease and Diagnosis. Pain Med..

[5] Burgstaller J.M., Porchet F., Steurer J., Wertli M.M. (2015). Arguments for the Choice of Surgical Treatments in Patients with Lumbar Spinal Stenosis—A Systematic Appraisal of Randomized Controlled Trials. BMC Musculoskelet. Disord..

[6] Lee B.H., Moon S.-H., Suk K.-S., Kim H.-S., Yang J.-H., Lee H.-M. (2020). Lumbar Spinal Stenosis: Pathophysiology and Treatment Principle: A Narrative Review. Asian Spine J..

[7] Byvaltsev V.A., Kalinin A.A., Hernandez P.A., Shepelev V.V., Pestryakov Y.Y., Aliyev M.A., Giers M.B. (2022). Molecular and Genetic Mechanisms of Spinal Stenosis Formation: Systematic Review. Int. J. Mol. Sci..

[8] Barker P.A., Mantyh P., Arendt-Nielsen L., Viktrup L., Tive L. (2020). Nerve Growth Factor Signaling and Its Contribution to Pain. J. Pain Res..

[9] Morel L., Domingues O., Zimmer J., Michel T. (2020). Revisiting the Role of Neurotrophic Factors in Inflammation. Cells.

[10] Skaper S.D., Skaper S.D. (2018). Neurotrophic Factors: An Overview. Neurotrophic Factors: Methods and Protocols.

[11] Skup M. (2018). Neurotrophins: Evolution of Concepts on Rational Therapeutic Approaches. Postep. Biochem..

[12] Sobańska M., Sobański D., Staszkiewicz R., Gogol P., Strojny D., Pawłaszek T., Dammerman W., Grabarek B.O. (2025). Modulation of Neurturin Expression by Lumbosacral Spinal Stenosis, Lifestyle Factors, and Glycemic Dysregulation. Biomedicines.

[13] Sobański D., Sobańska M., Staszkiewicz R., Strojny D., Grabarek B.O. (2025). Changes in the Expression Profile of Growth-Associated Protein 43 in Degenerative Lumbosacral Stenosis. J. Clin. Med..

[14] Sobański D., Bogdał P., Staszkiewicz R., Sobańska M., Filipowicz M., Czepko R.A., Strojny D., Grabarek B.O. (2024). Evaluation of Differences in Expression Pattern of Three Isoforms of the Transforming Growth Factor Beta in Patients with Lumbosacral Stenosis. Cell Cycle.

[15] Xiong H.-Y., Hendrix J., Schabrun S., Wyns A., Campenhout J.V., Nijs J., Polli A. (2024). The Role of the Brain-Derived Neurotrophic Factor in Chronic Pain: Links to Central Sensitization and Neuroinflammation. Biomolecules.

[16] Neurotrophin Family (2021). Handbook of Hormones.

[17] Abdolahi S., Zare-Chahoki A., Noorbakhsh F., Gorji A. (2022). A Review of Molecular Interplay between Neurotrophins and miRNAs in Neuropsychological Disorders. Mol. Neurobiol..

[18] Ateaque S., Merkouris S., Barde Y.-A. (2023). Neurotrophin Signalling in the Human Nervous System. Front. Mol. Neurosci..

[19] Bruno F., Abondio P., Montesanto A., Luiselli D., Bruni A.C., Maletta R. (2023). The Nerve Growth Factor Receptor (NGFR/p75NTR): A Major Player in Alzheimer’s Disease. Int. J. Mol. Sci..

[20] Shu Y.-H., Lu X.-M., Wei J.-X., Xiao L., Wang Y.-T. (2015). Update on the Role of p75NTR in Neurological Disorders: A Novel Therapeutic Target. Biomed. Pharmacother..

[21] Moghanlou A.E., Yazdanian M., Roshani S., Demirli A., Seydyousefi M., Metz G.A.S., Faghfoori Z. (2023). Neuroprotective Effects of Pre-Ischemic Exercise Are Linked to Expression of NT-3/NT-4 and TrkB/TrkC in Rats. Brain Res. Bull..

[22] Jackson A.R., Dhawale A.A., Brown M.D. (2015). Association between Intervertebral Disc Degeneration and Cigarette Smoking: Clinical and Experimental Findings. JBJS Rev..

[23] Elmasry S., Asfour S., de Rivero Vaccari J.P., Travascio F. (2015). Effects of Tobacco Smoking on the Degeneration of the Intervertebral Disc: A Finite Element Study. PLoS ONE.

[24] Chen Z., Li X., Pan F., Wu D., Li H. (2018). A Retrospective Study: Does Cigarette Smoking Induce Cervical Disc Degeneration?. Int. J. Surg..

[25] Kiraz M., Demir E. (2020). Relationship of Lumbar Disc Degeneration with Hemoglobin Value and Smoking. Neurochirurgie.

[26] Staszkiewicz R., Gładysz D., Gralewski M., Garczarek M., Gadzieliński M., Grabarek B.O. (2022). Pathomechanism of the IVDs Degeneration and the Role of Neurotrophic Factors and Concentration of Selected Elements in Genesis of Low Back Pain. Curr. Pharm. Biotechnol..

[27] An H., Liu Z., Li L., Xu Y., Fan G. (2023). Effects of Brain-Derived Neurotrophic Factor on Neuronal Activity, Pain, and Related Cytokines in Rats with Lumbar Spinal Stenosis. Chin. J. Tissue Eng. Res..

[28] Sobański D., Staszkiewicz R., Sobańska M., Strojny D., Grabarek B.O. (2025). Effects of Pain in Lumbosacral Stenosis and Lifestyle-Related Factors on Brain-Derived Neurotrophic Factor Expression Profiles. Mol. Pain.

[29] Zheng Q., Lin R., Wang D., Zheng C., Xu W. (2024). Effects of Circulating Inflammatory Proteins on Spinal Degenerative Diseases: Evidence from Genetic Correlations and Mendelian Randomization Study. JOR Spine.

[30] Dai W.-L., Yan B., Bao Y.-N., Fan J.-F., Liu J.-H. (2020). Suppression of Peripheral NGF Attenuates Neuropathic Pain Induced by Chronic Constriction Injury through the TAK1-MAPK/NF-κB Signaling Pathways. Cell Commun. Signal..

[31] Lin Y.-T., Ro L.-S., Wang H.-L., Chen J.-C. (2011). Up-Regulation of Dorsal Root Ganglia BDNF and trkB Receptor in Inflammatory Pain: An in Vivo and in Vitrostudy. J. Neuroinflamm..

[32] Staszkiewicz R., Gładysz D., Sobański D., Bolechała F., Golec E., Dammermann W., Grabarek B.O. (2024). The Impacts of Intervertebral Disc Degeneration of the Spine, Alcohol Consumption, Smoking Tobacco Products, and Glycemic Disorders on the Expression Profiles of Neurotrophins-3 and -4. Biomedicines.

[33] Varghese F., Bukhari A.B., Malhotra R., De A. (2014). IHC Profiler: An Open Source Plugin for the Quantitative Evaluation and Automated Scoring of Immunohistochemistry Images of Human Tissue Samples. PLoS ONE.

[34] Schroeder A.B., Dobson E.T.A., Rueden C.T., Tomancak P., Jug F., Eliceiri K.W. (2021). The ImageJ Ecosystem: Open-source Software for Image Visualization, Processing, and Analysis. Protein Sci..

[35] Khan N., Smith M.T. (2015). Neurotrophins and Neuropathic Pain: Role in Pathobiology. Molecules.

[36] Wijayanti I.A.S., Adnyana I.M.O., Widyadharma I.P.E., Wiratnaya I.G.E., Mahadewa T.G.B., Astawa I.N.M. (2024). Neuroinflammation Mechanism Underlying Neuropathic Pain: The Role of Mesenchymal Stem Cell in Neuroglia. AIMS Neurosci..

[37] Song Q., E S., Zhang Z., Liang Y. (2024). Neuroplasticity in the Transition from Acute to Chronic Pain. Neurotherapeutics.

[38] Galvez-Sánchez C.M., Montoro C.I., Duschek S., Reyes del Paso G.A. (2020). Depression and Trait-Anxiety Mediate the Influence of Clinical Pain on Health-Related Quality of Life in Fibromyalgia. J. Affect. Disord..

[39] Michaelides A., Zis P. (2019). Depression, Anxiety and Acute Pain: Links and Management Challenges. Postgrad. Med..

[40] Sharma D., Feng X., Wang B., Yasin B., Bekker A., Hu H., Tao Y.-X. (2024). NT-3 Contributes to Chemotherapy-Induced Neuropathic Pain through TrkC-Mediated CCL2 Elevation in DRG Neurons. EMBO Rep..

[41] Tonyan S., Pospelova M., Krasnikova V., Fionik O., Alekseeva T., Samochernykh K., Ivanova N., Vavilova T., Vasilieva E., Makhanova A. (2023). Neurotrophin-3 (NT-3) as a Potential Biomarker of the Peripheral Nervous System Damage Following Breast Cancer Treatment. Pathophysiology.

[42] Bonomini F., Favero G., Castrezzati S., Borsani E. (2023). Role of Neurotrophins in Orofacial Pain Modulation: A Review of the Latest Discoveries. Int. J. Mol. Sci..

[43] Sun X., Ni S., Zhou Q., Zou D. (2024). Exogenous NT-3 Promotes Phenotype Switch of Resident Macrophages and Improves Sciatic Nerve Injury through AMPK/NF-κB Signaling Pathway. Neurochem. Res..

[44] Li G., Zhang B., Sun J., Shi L., Huang M., Huang L., Lin Z., Lin Q., Lai B., Ma Y. (2021). An NT-3-Releasing Bioscaffold Supports the Formation of TrkC-Modified Neural Stem Cell-Derived Neural Network Tissue with Efficacy in Repairing Spinal Cord Injury. Bioact. Mater..

[45] Omar N.A., Kumar J., Teoh S.L. (2022). Neurotrophin-3 and Neurotrophin-4: The Unsung Heroes That Lies behind the Meninges. Neuropeptides.

[46] Guo W., Liu K., Wang Y., Ge X., Ma Y., Qin J., Zhang C., Zhao Y., Shi C. (2024). Neurotrophins and Neural Stem Cells in Posttraumatic Brain Injury Repair. Anim. Models Exp. Med..

[47] Gabryelska A., Turkiewicz S., Ditmer M., Sochal M. (2023). Neurotrophins in the Neuropathophysiology, Course, and Complications of Obstructive Sleep Apnea—A Narrative Review. Int. J. Mol. Sci..

[48] Staszkiewicz R., Sobański D., Bryś K., Och W., Garczarek M., Ulasavets U., Stasiowski M., Dammermann W., Strojny D., Grabarek B.O. (2023). Effect of Glycemic Disorders and Habits on the Concentration of Selected Neurotrophic Factors in Patients with Lumbosacral Intervertebral Disc Degeneration. Curr. Pharm. Biotechnol..

[49] Caliri A.W., Tommasi S., Besaratinia A. (2021). Relationships among Smoking, Oxidative Stress, Inflammation, Macromolecular Damage, and Cancer. Mutat. Res..

[50] Li Y., Xia B., Li R., Yin D., Wang Y., Liang W. (2017). Expression of Brain-Derived Neurotrophic Factors, Neurotrophin-3, and Neurotrophin-4 in the Nucleus Accumbens during Heroin Dependency and Withdrawal. Neuroreport.

[51] Chimbar L., Moleta Y. (2018). Naloxone Effectiveness: A Systematic Review. J. Addict. Nurs..

[52] Miller M.W., Mooney S.M. (2004). Chronic Exposure to Ethanol Alters Neurotrophin Content in the Basal Forebrain-Cortex System in the Mature Rat: Effects on Autocrine-Paracrine Mechanisms. J. Neurobiol..

[53] Requena-Ocaña N., Araos P., Flores M., García-Marchena N., Silva-Peña D., Aranda J., Rivera P., Ruiz J.J., Serrano A., Pavón F.J. (2021). Evaluation of Neurotrophic Factors and Education Level as Predictors of Cognitive Decline in Alcohol Use Disorder. Sci. Rep..

[54] Silva-Peña D., García-Marchena N., Alén F., Araos P., Rivera P., Vargas A., García-Fernández M.I., Martín-Velasco A.I., Villanúa M.Á., Castilla-Ortega E. (2019). Alcohol-Induced Cognitive Deficits Are Associated with Decreased Circulating Levels of the Neurotrophin BDNF in Humans and Rats. Addict. Biol..

[55] Yang J.-T., Chang C.-N., Wu J.H., Chung C.-Y., Weng H.-H., Cheng W.-C., Lee T.-H. (2008). Cigarette Smoking Decreases Neurotrophin-3 Expression in Rat Hippocampus after Transient Forebrain Ischemia. Neurosci. Res..

[56] Kimata H. (2004). Passive Smoking Elevates Neurotrophin Levels in Tears. Hum. Exp. Toxicol..

[57] Jayathilake N.J., Phan T.T., Kim J., Lee K.P., Park J.M. (2025). Modulating Neuroplasticity for Chronic Pain Relief: Noninvasive Neuromodulation as a Promising Approach. Exp. Mol. Med..

[58] Zhang F., Zhao X., Shen H., Zhang C. (2016). Molecular Mechanisms of Cell Death in Intervertebral Disc Degeneration (Review). Int. J. Mol. Med..

[59] Xiaogang M., Quanshan H., Liping Z., Kaken H. (2017). The Expression of Cytokine and Its Significance for the Intervertebral Disks of Kazakhs. J. Clin. Lab. Anal..

[60] Nakazawa K.R., Walter B.A., Laudier D.M., Krishnamoorthy D., Mosley G.E., Spiller K.L., Iatridis J.C. (2018). Accumulation and Localization of Macrophage Phenotypes with Human Intervertebral Disc Degeneration. Spine J..

[61] Bielewicz J., Daniluk B., Kamieniak P. (2022). VAS and NRS, Same or Different? Are Visual Analog Scale Values and Numerical Rating Scale Equally Viable Tools for Assessing Patients after Microdiscectomy?. Pain Res. Manag..

